# DNA hybridisation kinetics using single-molecule fluorescence imaging

**DOI:** 10.1042/EBC20200040

**Published:** 2021-04-16

**Authors:** Rebecca Andrews

**Affiliations:** Gene Machines Laboratory, Biological Physics Research Group, Clarendon Laboratory, Department of Physics, University of Oxford, Oxford, U.K.

**Keywords:** DNA hybridisation, Fluorescence, Single-Molecule

## Abstract

Deoxyribonucleic acid (DNA) hybridisation plays a key role in many biological processes and nucleic acid biotechnologies, yet surprisingly there are many aspects about the process which are still unknown. Prior to the invention of single-molecule microscopy, DNA hybridisation experiments were conducted at the ensemble level, and thus it was impossible to directly observe individual hybridisation events and understand fully the kinetics of DNA hybridisation. In this mini-review, recent single-molecule fluorescence-based studies of DNA hybridisation are discussed, particularly for short nucleic acids, to gain more insight into the kinetics of DNA hybridisation. As well as looking at single-molecule studies of intrinsic and extrinsic factors affecting DNA hybridisation kinetics, the influence of the methods used to detect hybridisation of single DNAs is considered. Understanding the kinetics of DNA hybridisation not only gives insight into an important biological process but also allows for further advancements in the growing field of nucleic acid biotechnology.

## Background

Deoxyribonucleic acid (DNA) hybridisation, especially of short DNAs, is an essential process in biology, however, much is still unknown about the exact process of hybridisation and its kinetics. As DNA hybridisation is also used across a range of biological and biotechnological applications such as hybridisation-based next-generation DNA sequencing [1], fluorescence-based *in situ* imaging [2], and super-resolution imaging [3,4], it is essential to achieve a much more complete understanding of the kinetics of hybridisation.

**Figure 1:**
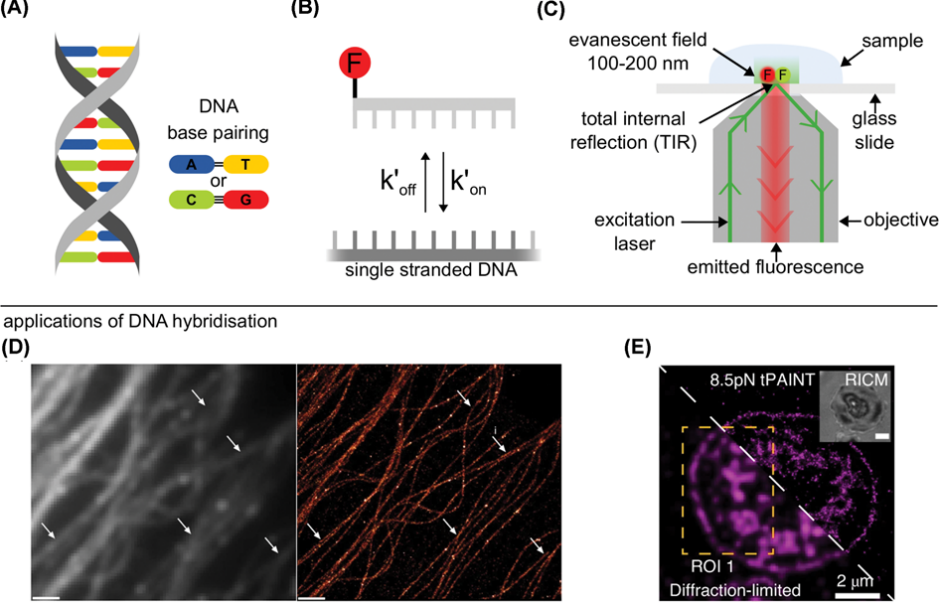
Single-molecule applications of transient DNA hybridisation (**A**) DNA exists as a double helix with DNA base pairing rules: adenine (A) - thymine (T) and cytosine (C) - guanine (G), two and three hydrogen bonds between pairs respectively (black lines). (**B**) Transient hybridisation can be characterised by the association rate (${\text{k}}_{\text{on}}^{\prime }$) as the DNA binds and the dissociation rate (${\text{k}}_{\text{off}}^{\prime }$) as the DNA unbinds. Top strand is fluorescently labelled (F). (**C**) Fluorophores are excited and fluorescence is collected for a single-molecule total internal reflection fluorescence (TIRF) microscope. Evanescent field, created by total internal reflection of incident laser beam, excites fluorophores close to the surface (100–200 nm). (**D**) Left: Diffraction limited image of microtubules imaged using the ensemble fluorescence produced by the transient hybridisation of labelled DNAs. Right: Super-resolved image of microtubules imaged using the transient hybridisation of single-labelled DNAs. White arrows indicated areas that are significantly enhanced by super-resolution imaging. Scale bar: 1 µm. (Reprinted from Jungmann et al., Nat. Methods, 2014; used with permission). (**E**) Bottom-half: diffraction limited image, top-half: strain-free tension-PAINT (sf-tPAINT) image of 8.5 pN integrin forces during platelet activation. Inset is a reflection interference contrast microscopy (RICM) image (Reprinted from Brockman et al., Nat. Methods, 2020: used with permission).

DNA hybridisation is not a necessarily permanent reaction with DNA able to undergo many reversible hybridisation events known as transient hybridisation. Transient DNA hybridisation occurs when a double-stranded DNA (dsDNA), see Figure 1A, is formed temporarily (for milliseconds to seconds) via base-pairing of two complementary strands; the dsDNA subsequently thermally dissociates into single-stranded DNAs (ssDNAs), see Figure 1B. Ensemble measurements have long been established to study DNA hybridisation [5] but lack the capability to directly observe heterogeneity of single DNAs.

Single-molecule fluorescence imaging excites only a small volume of a sample in order to reduce the diffuse background fluorescence, enabling single DNA molecules to be imaged. In general, single-molecule microscopy has many key advantages over ensemble microscopy such as the ability to independently measure the association rate and dissociation rate of a reaction and also the ability to observe static and dynamic heterogeneity within samples otherwise lost to averaging in ensemble measurements. A common single-molecule fluorescence microscopy technique is total internal reflection fluorescence (TIRF) microscopy (Figure 1C), where the excitation light is totally internally reflected at the boundary to a glass slide-mounted sample [6]. The resulting non-propagating exponentially decaying evanescent wave penetrates the sample 100–200 nm creating a small illumination volume, key for single-molecule microscopy. TIRF microscopy restricts spatially where biomolecules can be observed limiting reactions to, or just above, the surface of the glass slide, lending itself well to experiments with surface-immobilised molecules. A modification that can be made to traditional TIRF microscopes to improve the imaging of single-molecule surface-based DNA hybridisation is changing the intensity profile of the incident laser beam to a flat-top profile instead of a gaussian for even illumination of the field of view [7].

Single-molecule fluorescence microscopy allows individual DNA hybridisation events to be observed directly, therefore, enabling the investigation of the kinetics of such reactions. Commonly in surface-based studies, that use TIRF microscopy, ssDNAs are labelled with fluorescent dyes and recorded for seconds to minutes as they bind to a complementary ssDNAs appropriately spaced on a solid support. Transient hybridisation can be quantified as two separate processes, with binding of the nucleic acids described by an association rate, ${\text{k}}_{\text{on}}^{\prime }$, and the separation of the double-stranded nucleic acid described by a dissociation rate, ${\text{k}}_{\text{off}}^{\prime }$, see Figure 1B. For the reaction shown in Figure 1B, the association rate, ${\text{k}}_{\text{on}}^{\prime }$, is dependent on the concentration of the fluorescently labelled ssDNAs available for binding. Another way the binding can be quantified, taking into consideration the concentration of the ssDNAs, is by the association rate constant *k_on_* (M^−1^s^−1^). For labelled ssDNA concentrations below 300 nM, one can assume that there are no reactions between the labelled ssDNAs, making the dissociation rate, ${\text{k}}_{\text{off}}^{\prime }$, equivalent to the dissociation rate constant, *k_off_* (s^−1^) [8]. Further, under the assumption of no photobleaching of the fluorescent dyes, the association rate constant, *k_on_*, can be written as $k_{on}=\tau_{unbound}^{-1}{\left[ssDNA\right]}^{-1}$ and the dissociation rate constant, *k_off_*, can be written as $k_{off}=\tau_{bound}^{-1}$, where τ_unbound_ is the average time between hybridisation events and τ_bound_ is the average time of a hybridisation event [9].

Using various single-molecule fluorescence microscopy techniques, the transient binding of fluorescently labelled DNAs has been utilised to create biotechnologies such as DNA points accumulation for imaging in nanoscale topography (DNA-PAINT) [3]. DNA-PAINT uses repeated transient hybridisation of short fluorescently labelled DNA to an immobilised complementary DNA to create a super-resolved image, see Figure 1D, with the method being quantitative [10] and having the ability to create multicolour images [11,12], even to create 124-plex images within minutes [13]. Since its invention, DNA-PAINT has been implemented alongside many different super-resolution microscopy techniques in order to image targets inside cells, such as structured illumination microscopy (SIM) [14], stimulated emission depletion (STED) microscopy [4], stochastic optical reconstruction microscopy (STORM) [15,16] and spinning disk confocal (SDC) microscopy [17]. DNA-PAINT so far has a wide range of biological applications such as imaging synaptic proteins [18], imaging forces inside live cells [19] (Figure 1E), creating 3D images of internal cell structures [11,16] and immunostaining of neuronal cells, tissues and microtubules [4,14] (Figure 1D). For DNA-PAINT, and other biotechnologies, a greater understanding of the kinetics of DNA hybridisation will lead to the optimisation of hybridisation assays making these technologies more powerful.

## Single-molecule fluorescence microscopy techniques and hybridisation kinetics

### Surface-immobilised single-molecule DNA hybridisation

**Figure 2:**
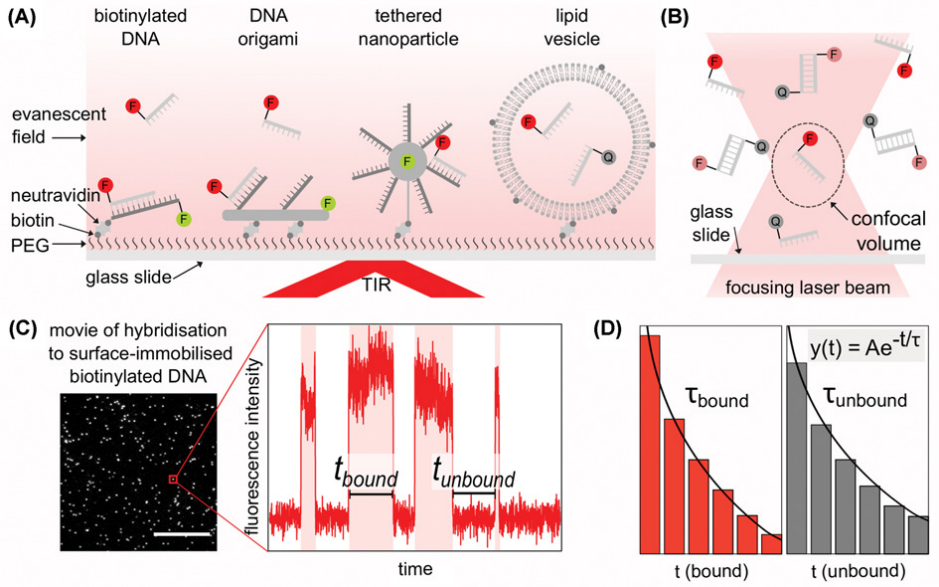
Single-molecule methods to measure DNA hybridisation kinetics (**A**) Methods for surface-immobilised single-molecule DNA hybridisation measurements. From left to right: single stranded DNA (ssDNA) immobilised via biotin/neutravidin, ssDNAs immobilised via DNA origami, ssDNAs immobilised via a tethered nanoparticle, ssDNAs confined via immobilised lipid vesicle. Polyethylene glycol (PEG) on surface of glass slide to allow immobilisation via biotin and neutravidin. In the sample, ssDNA labelled with fluorophores (F) or fluorescence quenchers (Q) are excited by an evanescent field from total internal reflection (TIR) of the incident laser beam. (**B**) Confocal microscopy for single-molecule imaging of hybridisation between fluorescently labelled ssDNAs and ssDNAs labelled with a quencher within the confocal volume (dashed ellipse). (**C**) Example of field of view from movie of hybridisation of fluorescently labelled ssDNAs to surface-immobilised DNA. Scale bar 10 µm. Red box: Example fluorescence intensity vs. time trace for a single unlabelled surface-immobilised DNA undergoing transient hybridisation with fluorescently labelled ssDNAs. A hybridisation event is characterised as a rise in fluorescence. The time for hybridisation can be measured as t_bound_ and the time between binding events measured as t_unbound_. (**D**) Histograms of t_bound_ and t_unbound_, fitted with a decaying exponential function (y(t) = Aexp(−t/τ), where A is a constant, τ is the calculated average time) to calculate the average bound time, τ_bound_ and the average unbound time τ_unbound_.

Commonly, surface-immobilised DNA hybridisation experiments are conducted using TIRF microscopy, where labelled or unlabelled ssDNAs (probes) hybridise with ssDNAs immobilised on a passivated glass surface. Immobilisation is usually achieved through a biotin–neutravidin linker; however, there are alternative ways to immobilise DNA on the surface, such as by using ssDNAs immobilised on DNA origami [20], on surface-tethered nanoparticles [21] or on long tethers [22], see Figure 2A. A large advantage of studying DNA hybridisation with immobilised DNA, is the ability to observe the same DNA molecule during multiple hybridisation events; however, the local environment for surface-immobilised DNA is drastically different.

From single-molecule fluorescence movies of surface-immobilised DNA the kinetics of hybridisation can be calculated through the changes in the intensity of fluorescence measured over time – the exact changes depend on the experimental fluorophore design. For example, a fluorophore can be used for localisation of a binding site either on the immobilised DNA, or in close proximity, and a differently coloured fluorophore can be directly excited on the hybridising DNA with a recorded increase in fluorescence upon hybridisation. A similar design uses a pair of fluorophores, one on each ssDNA, to observe binding through the use of Förster resonance energy transfer (FRET) between the two fluorophores in close proximity. Another method of fluorophore labelling, is labelling one set of the ssDNAs with a fluorescence quencher, a non-fluorescent molecule that absorbs energy, and when the quencher strand hybridises with a fluorescently labelled strand, the reduction/absence of fluorescence indicates binding. Figure 2C shows a field of view from a movie of labelled ssDNAs being directly excited when binding to surface-immobilised ssDNAs that were previously localised using a different coloured fluorophore, first example in Figure 2A. For each single-molecule a fluorescence intensity vs. time trace can be plotted for the length of the movie. By measuring the duration of binding events (t_bound_) and the time between binding events (t_unbound_) frequency histograms can be created and fitted by a decaying exponential function. From the decaying exponential fit, the average time of hybridisation (τ_bound_) can be calculated from the t_bound_ histogram and the average time between hybridisation events (τ_unbound_) can be calculated from the t_unbound_ histogram, as shown in Figure 2D.

Compared with solution-based experiments, ssDNAs that hybridise with surface-immobilised DNAs experience a greater repulsive electrostatic force [23–25]. When modelled, the electrostatic repulsion from surface-immobilised ssDNAs is the main reason for reduced *k_on_* when compared with solution-based models [26]. Crowding of the DNA at high densities [27] and non-specific interactions of the probes with the surface [28] also contribute to a reduction in *k_on_*. Intriguingly, single-molecule measurements have shown that after non-specific binding, probes perform a search process on the surface but the result is a low yield of hybridisation with the immobilised DNA, therefore, there is no increase in *k_on_* [27,29]. On the other side of the reaction, single-molecule surface-based studies are able to show *k_off_* is a combination of multiple distinct dissociation rates, corresponding to different dissociation behaviours, rather than a single average rate as seen in ensemble studies [29,30]. Interestingly, the type of surface used can also affect *k_off_* with hydrophilic surfaces decreasing the average *k_off_* when compared with hydrophobic surfaces [31].

There are alternative single-molecule fluorescence methods for surface-based experiments which do not directly tether the DNA, such as trapping or through confinement of the hybridising DNAs. Surface-tethered lipid vesicles (∼100 nm in diameter) which contain fluorescently labelled biomolecules are an example of confinement [32]; within each vesicle, the DNAs can freely diffuse and interact without being modified or hindered by tethering allowing DNA hybridisation observed [33], shown in Figure 2A. Since the membrane of lipid vesicles acts as a barrier to exchange molecules with the exterior of the vesicles, considerable effort has been taken to make them porous [34–36]. Even with such alternatives, there is no universal method of surface immobilisation which allows DNA hybridisation kinetics to be observed as they would be free in solution.

### Single-molecule DNA hybridisation in solution

Traditionally, ensemble fluorescence experiments of DNA hybridisation were conducted in solution; currently, this is also a method that makes this possible at the single-molecule level. Through the use of a confocal microscope (Figure 2B), a small volume of a sample (∼0.2 fl) [37] can be fluorescently illuminated, enabling imaging of single molecules as they diffuse in solution. However, standard confocal microscopy only provides a brief snapshot (∼1 ms) as each molecule randomly diffuses through the excitation volume. Therefore, standard confocal microscopy is not well suited to observing multiple hybridisation events on a single molecule over long time periods, which is needed to gather data on hybridisation kinetics.

A new confocal microscopy method, 3D single-molecule tracking (3D-SMT), has been developed to observe transient DNA hybridisation in solution over longer time periods (∼1 s) [38]. As the total maximum acquisition time is short, only fast transient hybridisation events between very short DNAs (8 nucleotides or less) can be recorded. Another novel approach for longer observations of DNA hybridisation in solution uses fluorescence to monitor the position of a labelled DNA whilst in the presence of an electric field. The changes in the DNA diffusion and drift reveals the kinetics of hybridisation [39]. Clearly, measuring DNA hybridisation in solution would avoid the changes in the kinetic rates seen for surface immobilised assays, but currently there is no solution-based method able to provide as higher throughput on hybridisation kinetics over longer periods of time as surface-immobilised assays.

### Effect of fluorophores on hybridisation kinetics

DNA hybridisation can simply be modelled as the interaction of two ssDNAs, however, in fluorescence microscopy one or both of the DNAs is modified for the inclusion of a small-molecule fluorescent dye. Fluorescent dyes are usually chosen specifically for their optical properties with less consideration for how the fluorophore interacts locally. When using an assay with fluorescently labelled DNAs, dye–DNA and dye–dye interactions during DNA hybridisation can potentially alter hybridisation kinetics.

At the ensemble level, dye–dye or dye–quencher interactions, where a quencher is a non-fluorescent molecule that absorbs energy, between labelled strands have been shown to reduce *k_off_*, therefore, creating a stabilising effect for hybridisation [40,41]. As well as dye–dye interactions, dyes attached at the end of a DNA, also known as terminal dyes, reduce *k_off_* due to stacking interactions with the adjacent end DNA bases [42].

Single-molecule fluorescence measurements have been able to look further at the changes in hybridisation kinetics due to the presence of fluorophores. DNA hybridisation between two fluorescently labelled ssDNAs, or within a fluorescently labelled DNA hairpin, found dye–dye interactions to increase the stability of the hybridisation and reduce *k_off_* [33,43]. Similarly, single-molecule experiments show terminal labelling with a cyanine fluorescent dye (Cy3) stabilises hybridisation by stacking with terminal bases, reducing *k_off_*, but also leads to an increase in *k_on_* of oligonucleotides [33,44,45]. Terminal dyes not only stabilise hybridisation through stacking but electrostatically interact with the DNA in dye–DNA interactions, with positively charged dyes showing a larger stabilising effect than negatively charged dyes [43]. Fluorescently labelling DNA clearly shows stabilising effects during hybridisation leading to a decreased *k_off_* but can also increase *k_on_* in terminally labelled DNA. Therefore, the transient binding of fluorescently labelled ssDNAs needs to be considered a modified interaction compared with unlabelled DNAs when analysing hybridisation kinetics but still acts as an incredibly powerful and useful tool for biotechnological applications.

## Intrinsic factors affecting hybridisation kinetics

### DNA sequence

A DNA duplex exists as two ssDNAs bound together through complementary base binding via hydrogen bonds and stacking interactions between bases. For base-pairing, adenine (A)–thymine (T) binding consists of two hydrogen bonds and is, therefore, weaker than guanine (G)–cytosine (C) binding which forms three hydrogen bonds, see Figure 1A. It is well known that a DNA sequence with a higher G/C content will hybridise more stably than a sequence containing mostly A/T bases, consequently, playing a determining factor in the dissociation rate of transient hybridisation. Intriguingly, modelling of sequence-dependent effects on DNA hybridisation shows that DNA sequence can, in fact, affect both the association and dissociation rates [46]. At the single-molecule level, where DNA hybridisation kinetics can be directly imaged, the DNA sequence used for multiple binding sites has been seen to affect the *k_on_* of individual probes. If multiple side-by-side binding sequences are used, there is a linear increase in the total number of binding events to that entire binding site compared with a single binding sequence, as would be logically expected. If the binding sequence is designed as periodic sequence motifs, such as a repeating TCC sequence, rather than the whole binding sequence repeating side-by-side, the same linear increase in *k_on_* with an increase in the number of binding sites is observed for the entire binding area [47]. Compared with side-by-side repeats, periodic sequence motifs provide the same number of individual binding sites, however, they are not all available at the same time due to the overlapping nature of the binding sites. Therefore, periodic sequence motifs increase *k_on_* for individual probes as predicted by theory, as there are more ways to correct a hybridisation misalignment before hybridisation of the entire probe [46].

### Number of bases

The actual sequence of bases is important in the hybridisation kinetics but also is the number of bases involved in hybridisation. From ensemble experiments, it is well established that long complementary ssDNAs bind more stably than short complementary ssDNAs during DNA hybridisation. Single-molecule experiments, are able to give greater insight into the exact changes in the kinetics of DNA hybridisation when the length of the DNA is changed. For short DNAs (7–12 nucleotides), an extra base in length can dramatically change the duration of time for which the ssDNA hybridises before it thermally dissociates [8,48,49], with *k_off_* drastically decreasing with increasing length [3,8,50,51]. In fact, there is a negative single exponential relationship between *k_off_* and the length of DNA [8]. However, during such hybridisation events, *k_on_* does not show any noticeable change when the ssDNA is free of secondary structures. Understanding the changing kinetics of DNA hybridisation at the level of an additional base is essential for the development of biotechnologies which depend on the repeated binding of short ssDNAs.

### Single base-pair mismatch

Although the length of a DNA can be an indication of the stability of its binding during hybridisation it is necessary to know the DNA sequence and its complementarity with the other DNA involved in hybridisation. Base-pair mismatches have a destabilising effect on DNA hybridisation, and the shorter the DNA the larger the effect of even a single base mismatch on the stability. Single-molecule experiments are able to show clearly that a single base-pair mismatch can dramatically decrease the time a DNA is hybridised due to increases in *k_off_* [48,52–55]. Commonly, single-molecule fluorescence experiments use a central mismatch for discrimination from the complementary [52–54,56], with a minority of experiments placing mismatches away from the centre [48,55]. Further, the type of mismatch can also affect the change in the dissociation rate; e.g. C–C mismatches are more destabilising than G–G mismatches [33]. The effect of mismatches on *k_on_* is much less clear. Multiple studies have found that *k_on_* decreases in the case of a single-base mismatch [33,52–54]; however, other studies claim there to be no change [55], or in fact, an increase [33]. When looking specifically at *k_on_* of short DNAs, seven contiguous bases are necessary for the smallest decrease in *k_on_*, indicating terminal mismatches have the least impact on *k_on_* [33].

### Secondary structure of ssDNA

Formation of a secondary structure due to transient hybridisation within a single DNA strand can occur when it diffuses in solution or when it is tethered on a solid support; such secondary structures can affect hybridisation kinetics. Specifically, both ensemble and simulated DNA hybridisation experiments showed that secondary structures in DNAs can drastically reduce *k_on_* during hybridisation [57–61]. Single-molecule experiments, which are capable of measuring small distances such as between two points on a DNA, are well suited to study the negative effect of secondary structures on *k_on_*. A reduction in *k_on_* is observed for secondary structures due to the structure of the hybridising strand, such as non-competing duplex regions within the strand [62], and also due to strands internally forming hairpins. When comparing DNAs of the same hybridising sequence those which formed hairpin secondary structures had a reduced *k_on_* compared with unstructured DNAs of a similar length [31]. This decrease in *k_on_*, due to secondary structures, can even be seen for short DNAs [22,63]. For technological applications, where fast repeated hybridisation is desired such as DNA PAINT [3], a reduced *k_on_* is unfavourable. To avoid this, a DNA sequence with no internal complementary bases can be used to eliminate most secondary structures that reduce *k_on_* [64].

## External factors affecting hybridisation kinetics

### Salts

The overall negative charge that nucleic acids carry, due to the negatively charged phosphate groups in the DNA backbone, results in repulsive electrostatic forces during DNA hybridisation which must be overcome for successful hybridisation. This repulsion applies to nucleic acids in solution but even more so to a situation where a local field is produced, such as nucleic acids immobilised on a surface – a key method used for single-molecule fluorescence DNA hybridisation studies.

A way to shield (or ‘screen’) the electrostatic forces, and consequently increase the rate of hybridisation, is to add cations in the form of salts to the local environment. Monovalent and divalent cations, such as Na^+^ and Mg^2+^, can be used to shield the electrostatic repulsion between two ssDNAs, hence, facilitating and stabilising hybridisation. At the ensemble level, which cation or combination of cations is the most effective is debated [25,65,66] but it is universally recognised that cations play a large role in shielding the electrostatic repulsion between ssDNAs, hence, encouraging duplex formation.

As one of the key controllable variables in single-molecule DNA hybridisation, the effects of salt type and concentration have been examined extensively. It is generally seen that *k_on_* positively correlates with the salt concentration in solution independent of which salt cation [8,22,31,33,64]. As an alternative, there are also other molecules that can provide electrostatic shielding such as cationic conjugated polymers (CCPs) [54]. In terms of hybridisation, the increased *k_on_* in the presence of cations is due to increasing the proportion of successful binding events [31] as the cations alter the structure of the ssDNAs making hybridisation more favourable [8]. This trend in increasing *k_on_*, however, is not seen for structured ssDNAs such as hairpins [31], as the opening rate of hairpins slows with increasing salt concentrations due to stabilisation of the secondary structure [67]. Although it is clear that an increase in salt concentration has a positive effect on *k_on_* for unstructured ssDNAs, any correlation with *k_off_* is not. Some studies report a slight decrease in *k_off_* with an increase in the salt concentration [8]; however, more commonly there is very little or no change in *k_off_* with increasing salt concentrations [22,31,33].

### Ethylene carbonate

Another way to affect hybridisation kinetics is by introducing ethylene carbonate (EC) an aprotic solvent thought to increase the solubility of the DNA bases [68]. Studied at the single-molecule level, in the presence of 0–15% EC, there is a five to tenfold decrease in *k_off_* in the transient binding of a 9-nucleotide probe. Intriguingly, there is no reduction in *k_on_* across the range of EC concentrations.

### Presence of DNA-binding proteins

Rapid transient binding of ssDNAs in biotechnologies that implement DNA transient hybridisation is key, for example, in creating super-resolution images using DNA PAINT [3]. A novel way to increase *k_on_* for such reactions is the use of Argonaute proteins that bind the ssDNA probes, providing them with a helical structure, before the probes hybridise [69]. The helical structure of the probes allows DNA hybridisation to occur at a faster rate – an order of magnitude increase in *k_on_*. Also, the presence of the protein is shown to stabilise binding decreasing *k_off_* significantly when compared with similar probes in the absence of Argonaute proteins.

### Concentration

An ever-present external factor in DNA hybridisation is the concentration of the ssDNAs in solution. Single-molecule studies show that the association rate, $k_{on}^{\prime }$, linearly increases with increasing concentration of hybridising ssDNAs in solution [3,8,44,63,70]. On the other side of the reaction, there is no known dependence between the dissociation rate $k_{off}^{\prime }$ and the concentration of the same ssDNAs in solution [3,63]. The maximum concentration of labelled ssDNAs in single-molecule TIRF microscopy measurements is limited to 50–100 nM, due to the fluorescence background produced by unbound DNA which if too high can prevent the detection of single molecules.

### Temperature

Another external factor during DNA hybridisation is the temperature, and as transient hybridisation is thermally driven, it is obvious that the temperature of the measurement will affect the kinetics DNA hybridisation. A high temperature provides short DNAs with more thermal energy to escape a hybridised state, as shown in the observed increase in *k_off_* in single-molecule imaging experiments [3], and when investigated further with single molecules the behaviour can described by Eyring transition state theory [71]. Interestingly, *k_on_* has been seen to slightly decrease with increasing temperature [3], possibly due to increased events where the ssDNA dissociates whilst in the process of trying to hybridise – known as abortive hybridisation.

## Concluding remarks and the future

Single-molecule fluorescence studies of DNA hybridisation, mainly of short DNAs (7–12 nucleotides), show that the association rate constant of hybridisation, *k_on_*, is clearly influenced by external factors such as salt concentration, DNA-binding proteins and temperature but also by secondary structures in the ssDNA. On the other hand, the dissociation rate constant of hybridisation, *k_off_*, is more clearly influenced by the length of the ssDNA and complementarity of the ssDNA. Further, one cannot disregard that the hybridisation kinetics measured using single-molecule fluorescence microscopy methods are affected by the methods needed to image single molecules, such as surface-immobilisation and labelling the DNAs with fluorophores. Studies on surface-immobilised molecules show an altered *k_on_* due to electrostatic repulsion and crowding at the surface; further, the presence of fluorescent dyes on labelled DNA decreases *k_off_* due to dye interactions that in turn increase the stability of the dsDNA formed. Such a complex dependency on many factors complicates the accurate prediction of the exact rates of hybridisation for chosen DNAs. With the ever popular and increasing use of nucleic acid hybridisation for biotechnologies, a greater insight into the kinetics of DNA hybridisation will not only allow for improvements of existing technologies but also the invention of many more.

## Summary

- DNA hybridisation is a key for biological functions but also in many biotechnologies.
- Single-molecule fluorescence experiments allow hybridisation kinetics to be directly imaged.
- Although not fully understood, there are many intrinsic and extrinsic factors that can affect DNA hybridisation kinetics.
- A more informed picture of the kinetics of DNA hybridisation will allow for greater advancements in nucleic acid-based biotechnologies.

## Open Access

Open access for this article was enabled by the participation of University of Oxford in an all-inclusive *Read & Publish* pilot with Portland Press and the Biochemical Society under a transformative agreement with JISC.

## Abbreviations

DNA: deoxyribonucleic acid
dsDNA: double-stranded DNA
DNA-PAINT: DNA Points Accumulation for Imaging in Nanoscale Topography
EC: ethylene carbonate
${\text{k}}_{\text{on}}^{\prime }$: association rate
${\text{k}}_{\text{off}}^{\prime }$: dissociation rate
*k_on_*: association rate constant
*k_off_*: dissociation rate constant
ssDNA: single-stranded DNA
TIRF: total internal reflection fluorescence
